# Comparison of positive detection rates between fluorescent staining and traditional KOH wet mount methods in the diagnosis of superficial fungal skin infections and analysis of factors associated with missed diagnosis

**DOI:** 10.3389/fmed.2026.1911614

**Published:** 2026-08-26

**Authors:** Li Zhou, Lihua Hu, Lu Pan, Feng Lin, Jia Huang, Yifan Tao, Mingqin Lu, Bo Yang, Dan Li

**Affiliations:** Department of Laboratory, Zhejiang Dermatology Hospital, Huzhou, Zhejiang, China

**Keywords:** calcofluor white, dermatophytosis, diagnosis, fluorescent staining, KOH wet mount, missed diagnosis, superficial fungal infection

## Abstract

**Background:**

Superficial fungal skin infections represent one of the most prevalent dermatological conditions worldwide, necessitating accurate and rapid laboratory diagnosis for appropriate therapeutic management.

**Objective:**

This retrospective study aimed to compare the positive detection rates between fluorescent staining and traditional KOH wet mount methods in patients with clinically suspected superficial fungal infections and to identify factors associated with missed diagnosis.

**Methods:**

A total of 151 patients with clinically suspected superficial fungal skin infections presenting to our dermatology department between January 1, 2025, and June 30, 2025, were enrolled. All specimens underwent parallel examination using both fluorescent staining (calcofluor white) and traditional 10% KOH wet mount methods. Diagnostic performance parameters including sensitivity, specificity, positive predictive value (PPV), and negative predictive value (NPV) were calculated. Multivariate logistic regression analysis was performed to identify independent risk factors for missed diagnosis.

**Results:**

The fluorescent staining method demonstrated a significantly higher positive detection rate (78.81%, 119/151) compared to the KOH wet mount method (58.28%, 88/151) (χ^2^ = 15.73, *P* < 0.001). Using fungal culture as the gold standard, fluorescent staining achieved a sensitivity of 94.35% versus 73.39% for KOH (*P* < 0.01), with specificities of 91.67 and 95.83%, respectively. Multivariate analysis revealed that prior antifungal treatment (OR = 3.84, 95% CI: 1.62–9.11, *P* = 0.002), inadequate specimen collection (OR = 2.76, 95% CI: 1.18–6.45, *P* = 0.019), and nail specimen type (OR = 2.31, 95% CI: 1.04–5.13, *P* = 0.039) were independent risk factors for missed diagnosis with KOH wet mount examination.

**Conclusion:**

Fluorescent staining provides superior diagnostic performance compared to traditional KOH wet mount for superficial fungal infections, with significantly higher sensitivity and comparable specificity.

## 1 Introduction

Superficial fungal infections constitute a substantial proportion of dermatological consultations globally, affecting an estimated 20–25% of the world’s population, with a global prevalence of approximately 25% and an increasing trend observed in recent decades (1). These infections, caused primarily by dermatophytes belonging to the genera *Trichophyton*, *Microsporum*, and *Epidermophyton*, alongside yeasts such as Candida and Malassezia species, manifest as various clinical entities including tinea corporis, tinea pedis, tinea cruris, tinea capitis, onychomycosis, and pityriasis versicolor (2). The global burden of superficial mycoses has increased substantially over recent decades, attributable to factors including population aging, rising prevalence of immunocompromising conditions, widespread use of broad-spectrum antibiotics, and lifestyle changes that promote fungal transmission (3). The economic impact extends beyond direct medical costs to include lost productivity, reduced quality of life, and psychological distress associated with chronic or recurrent infections (4). Particularly concerning is the emergence of antifungal resistance among dermatophytes, which underscores the critical importance of accurate diagnosis to guide appropriate therapeutic interventions (5).

The diagnosis of superficial fungal infections traditionally relies on a combination of clinical examination and laboratory confirmation, with the potassium hydroxide (KOH) wet mount preparation serving as the cornerstone of mycological diagnosis for over a century (6). This technique involves treating clinical specimens—typically skin scrapings, hair, or nail clippings—with 10–20% KOH solution, which dissolves keratin and cellular debris while leaving fungal elements intact, thereby facilitating their visualization under light microscopy (7). The KOH wet mount method offers several advantages, including low cost, rapid turnaround time, minimal equipment requirements, and widespread availability, making it particularly suitable for resource-limited settings (7). However, this technique suffers from significant limitations that compromise its diagnostic utility. The sensitivity of KOH examination varies widely in published literature, ranging from 33.7 to 93% depending on the specimen type, examiner experience, and clearing time (8). The absence of staining in conventional KOH preparations results in low contrast between fungal elements and background material, rendering interpretation challenging and highly dependent on observer expertise (9).

Fluorescent staining techniques have emerged as a promising advancement in the direct microscopic diagnosis of fungal infections, addressing many of the shortcomings inherent to conventional KOH examination (10). Calcofluor white (CFW), a fluorescent brightener originally developed for the textile industry, binds specifically to β-1,3 and β-1,4 polysaccharides, including chitin and cellulose, which are principal structural components of fungal cell walls (11). When exposed to ultraviolet or short-wavelength visible light, calcofluor white-stained fungal elements emit bright blue-green or apple-green fluorescence against a dark background, creating a stark contrast that substantially facilitates detection and reduces the interpretive burden on the examiner (12). This enhanced visualization capability theoretically translates to improved sensitivity and reduced interobserver variability compared to unstained KOH preparations. Additionally, fluorescent staining can be combined with KOH treatment to simultaneously achieve specimen clearing and fungal element highlighting, thereby integrating the benefits of both approaches (13). Several studies have demonstrated the superior diagnostic performance of fluorescent staining over conventional KOH examination, with reported sensitivities approaching 95–97% in certain clinical contexts (14).

Despite the theoretical advantages and encouraging preliminary data supporting fluorescent staining, its implementation in routine clinical practice remains inconsistent, and comprehensive comparative evaluations against traditional methods in diverse patient populations are limited (6). Several factors may contribute to the variable adoption of this technology, including the requirement for fluorescence microscopy equipment, higher reagent costs, and the need for specialized training in fluorescent specimen interpretation (7). Furthermore, while the enhanced detection capability of fluorescent staining is well-documented, the clinical significance of identifying fungal elements that would have been missed by conventional methods remains incompletely characterized. It is conceivable that some positive results obtained exclusively through fluorescent examination may represent colonization rather than true infection, or may detect non-viable organisms in patients who have received antifungal therapy (7). Conversely, missed diagnoses due to suboptimal sensitivity of conventional methods may result in inappropriate treatment, disease progression, and continued transmission (8).

A critical gap in the existing literature pertains to the systematic identification and characterization of factors that predispose to diagnostic failure with conventional microscopic methods (9). Understanding why certain specimens yield false-negative results with KOH examination is essential for optimizing specimen collection techniques, refining diagnostic algorithms, and identifying patient subgroups who may particularly benefit from fluorescent staining (10). Potential contributors to missed diagnosis include inadequate specimen quantity or quality, prior antifungal treatment reducing fungal burden, specimen type characteristics, disease duration, and examiner-dependent factors (11). However, rigorous multivariate analyses that control for confounding variables and quantify the independent contributions of these factors are scarce in the published literature (12).

The present study was designed to address these knowledge gaps through a comprehensive retrospective analysis comparing the diagnostic performance of fluorescent staining and traditional KOH wet mount methods in a contemporary cohort of patients with clinically suspected superficial fungal skin infections (13). This study provides three distinctive contributions to the existing literature: (1) unlike previous investigations that largely focused on single specimen types (e.g., nails alone) or single pathogens, we performed parallel comparisons across diverse specimen types (skin, nails, and hair) under a unified clinical protocol; (2) we employed multivariate logistic regression analysis to quantify the independent contributions of multiple risk factors for KOH diagnostic failure, whereas prior studies primarily used univariate analyses without adjusting for confounding variables; and (3) we are the first to simultaneously evaluate prior antifungal therapy, specimen adequacy, and specimen type as independent predictors of missed diagnosis in a Chinese population, providing directly actionable evidence for clinical practice. Our primary objectives were to: (1) determine and compare the positive detection rates of fluorescent staining versus conventional KOH examination; (2) calculate diagnostic performance parameters including sensitivity, specificity, positive and negative predictive values using fungal culture as the reference standard; and (3) identify independent risk factors associated with missed diagnosis through multivariate logistic regression analysis (14). We hypothesized that fluorescent staining would demonstrate significantly higher sensitivity compared to KOH wet mount while maintaining acceptable specificity, and that specific clinical and specimen-related factors would emerge as significant predictors of diagnostic failure with conventional methods. The findings from this investigation are anticipated to inform evidence-based recommendations for the selection of optimal diagnostic approaches in different clinical scenarios and patient populations.

## 2 Methodology

### 2.1 Study design and setting

This retrospective cross-sectional study was conducted at the Department of Dermatology and Venereology, Zhejiang Dermatology Hospital, a tertiary care academic medical center, between January 1, 2025, and June 30, 2025. The study protocol was approved by the Institutional Review Board (IRB Approval Number: LL2021-2) and conducted in accordance with the principles outlined in the Declaration of Helsinki. Due to the retrospective nature of the study and use of de-identified clinical data, the requirement for individual informed consent was waived by the ethics committee.

### 2.2 Study population

The study enrolled consecutive patients presenting to the dermatology outpatient clinic with clinical suspicion of superficial fungal skin infection. Inclusion criteria were: (1) age ≥ 18 years; (2) clinical presentation suggestive of superficial fungal infection based on dermatological examination, including but not limited to annular erythematous scaly plaques, interdigital maceration, nail discoloration or dystrophy, and hypopigmented or hyperpigmented patches; (3) availability of parallel microscopic examination results using both fluorescent staining and KOH wet mount methods; and (4) complete clinical and demographic information in medical records. Exclusion criteria included: (1) systemic antifungal therapy within 4 weeks or topical antifungal treatment within 2 weeks prior to specimen collection; (2) concurrent systemic immunosuppressive therapy; (3) known HIV infection or other primary immunodeficiency; (4) specimens from mucosal sites or deep tissue; and (5) pregnant or lactating women.

Sample size calculation was performed using the formula for comparison of two proportions. Based on previous literature reporting KOH sensitivity of approximately 60% and fluorescent staining sensitivity of approximately 85%, with α = 0.05 (two-sided) and β = 0.20 (power = 80%), the minimum required sample size was calculated as 138 subjects. Accounting for potential incomplete data or dropout, we targeted enrollment of 150 patients, and ultimately included 151 patients who met all eligibility criteria.

### 2.3 Specimen collection

All specimen collection procedures were performed by trained dermatology residents or attending physicians following standardized protocols. Prior to collection, the affected area was cleaned with 70% isopropyl alcohol to reduce bacterial contamination and allowed to air dry. For skin lesions, scrapings were obtained from the active advancing edge of the lesion using a sterile surgical blade held at a 45-degree angle. For nail specimens, subungual debris was collected from the proximal portion of the affected nail bed after trimming the distal nail plate, and nail clippings were obtained when appropriate. For scalp lesions, affected hairs were epilated using sterile forceps along with associated scales. Each specimen was divided into three portions: two portions for immediate microscopic examination (fluorescent staining and KOH wet mount) and one portion for fungal culture. Specimen adequacy was assessed subjectively by the collecting physician and documented as adequate, borderline, or inadequate based on quantity and quality of material obtained.

### 2.4 Laboratory methods

For KOH wet mount examination, specimens were placed on a clean glass slide, covered with 1–2 drops of 10% potassium hydroxide solution, and overlaid with a coverslip. Slides were allowed to clear for 15–30 min at room temperature or gently heated to accelerate clearing. Examination was performed under a standard light microscope at 100× and 400× magnification by trained laboratory technicians blinded to clinical information. A positive result was defined as the presence of septate hyphae, arthroconidia, or budding yeast cells consistent with fungal infection.

For fluorescent staining examination, a separate specimen aliquot was placed on a glass slide and treated with a combined reagent containing 0.1% calcofluor white M2R, 0.01% Evans blue counterstain, and 10% KOH. After application of a coverslip and 5–10 min of clearing time, slides were examined under a fluorescence microscope equipped with a UV excitation filter (excitation 355–425 nm, barrier filter 460 nm) at 100× and 400× magnification. Fungal elements appeared as bright blue-green fluorescent structures against a dark or reddish background. All microscopic examinations were performed within 2 h of specimen collection by experienced mycology technicians who were blinded to the results of the alternate method.

Fungal culture served as the reference standard for calculating sensitivity and specificity. Specimens were inoculated onto Sabouraud dextrose agar (SDA) with chloramphenicol and gentamicin to inhibit bacterial growth, and onto SDA with cycloheximide (Mycosel agar) to inhibit saprophytic fungi. Cultures were incubated at 25 and 37°C and examined weekly for up to 4 weeks. Identification of dermatophytes and yeasts was based on colony morphology, microscopic characteristics (lactophenol cotton blue preparations), and biochemical testing when necessary. For yeast species identification, we employed a combination of phenotypic methods: (1) colony color and morphology on CHROMagar Candida differential medium; (2) microscopic examination for blastoconidia and pseudohyphae morphology; and (3) carbohydrate assimilation profiles using the API 20C AUX system (bioMérieux, Marcy-l’Étoile, France) according to the manufacturer’s instructions.

### 2.5 Data collection

Clinical and laboratory data were extracted from electronic medical records using a standardized data collection form. Variables collected included: demographic information (age, sex); clinical characteristics (affected site, clinical diagnosis, duration of symptoms, prior antifungal treatment); specimen characteristics (specimen type, specimen adequacy assessment); and laboratory results (KOH examination result, fluorescent staining result, culture result with species identification when positive). All data extraction was performed by two independent investigators, with discrepancies resolved by consensus review.

### 2.6 Statistical analysis

All statistical analyses were performed using IBM SPSS Statistics version 26.0 (IBM Corporation, Armonk, NY, United States). Continuous variables were assessed for normality using the Shapiro-Wilk test and presented as mean ± standard deviation (SD) for normally distributed data or median with interquartile range (IQR) for non-normally distributed data. Categorical variables were expressed as frequencies and percentages. Comparison of positive detection rates between the two diagnostic methods was performed using McNemar’s test for paired proportions. Diagnostic performance parameters including sensitivity, specificity, positive predictive value (PPV), negative predictive value (NPV), and overall accuracy were calculated using fungal culture as the reference standard, with 95% confidence intervals (CI) derived using the Wilson score method. Univariate analysis of factors associated with missed diagnosis by KOH examination was conducted using chi-square test or Fisher’s exact test for categorical variables and independent *t*-test or Mann-Whitney U test for continuous variables. Variables with *P* < 0.10 in univariate analysis were entered into a multivariate binary logistic regression model using the enter method to identify independent risk factors, with results expressed as odds ratios (OR) and 95% CI. Receiver operating characteristic (ROC) curve analysis was performed to assess the predictive capacity of the logistic regression model. A two-tailed *P* < 0.05 was considered statistically significant for all analyses.

## 3 Results

### 3.1 Baseline characteristics

**FIGURE 1:**
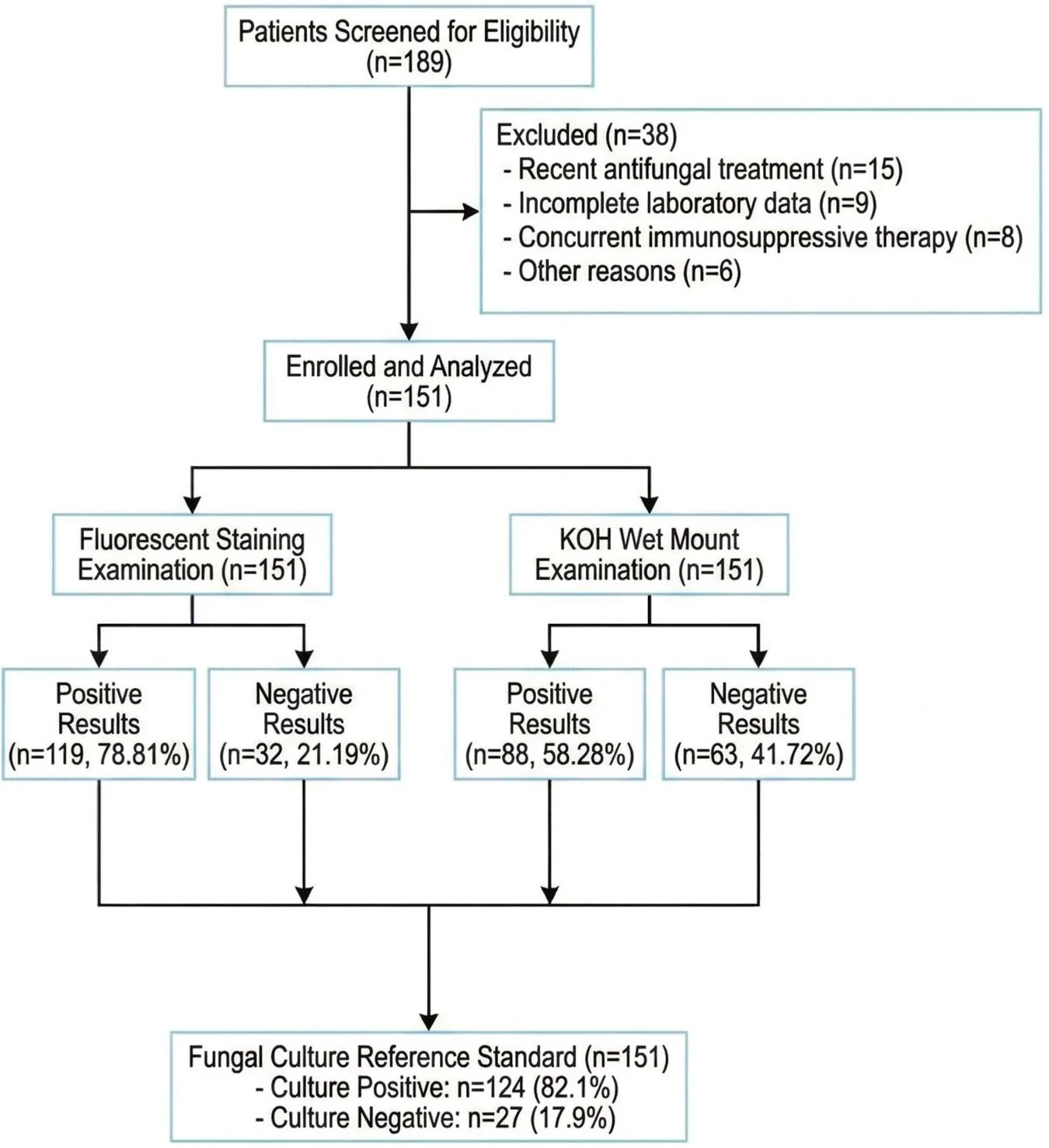
Flowchart of patient inclusion and exclusion criteria following CONSORT guidelines. The diagram illustrates the screening, enrollment, and analysis process for the 151 patients included in the final study cohort.

A total of 189 patients were initially screened for eligibility during the study period. Of these, 38 were excluded: 15 due to recent antifungal treatment, 9 due to incomplete laboratory data, 8 due to concurrent immunosuppressive therapy, and 6 for other reasons. The final study cohort comprised 151 patients who met all inclusion criteria (Figure 1). The demographic and clinical characteristics of the study population are summarized in Table 1. The mean age was 43.7 ± 15.3 years (range: 18–78 years), with a slight male predominance (54.3%, *n* = 82). The most common clinical presentations were tinea corporis (31.1%, *n* = 47), onychomycosis (25.2%, *n* = 38), tinea pedis (19.2%, *n* = 29), pityriasis versicolor (13.2%, *n* = 20), and tinea cruris (11.3%, *n* = 17). The median duration of symptoms prior to presentation was 8 weeks (IQR: 4–24 weeks). Twenty-three patients (15.2%) reported prior antifungal treatment that did not meet exclusion criteria. Specimen adequacy was rated as adequate in 118 cases (78.1%), borderline in 25 cases (16.6%), and inadequate in 8 cases (5.3%).

**TABLE 1:** Baseline demographic and clinical characteristics of study participants (*N* = 151).

Variable	Value
Age (years), mean ± SD	43.7 ± 15.3
Sex, n (%)	
Male	82 (54.3)
Female	69 (45.7)
Clinical diagnosis, n (%)	
Tinea corporis	47 (31.1)
Onychomycosis	38 (25.2)
Tinea pedis	29 (19.2)
Pityriasis versicolor	20 (13.2)
Tinea cruris	17 (11.3)
Symptom duration (weeks), median (IQR)	8 (4–24)
Prior antifungal treatment, n (%)	23 (15.2)
Specimen adequacy, n (%)	
Adequate	118 (78.1)
Borderline	25 (16.6)
Inadequate	8 (5.3)

SD, standard deviation; IQR, interquartile range.

### 3.2 Comparison of positive detection rates

**FIGURE 2:**
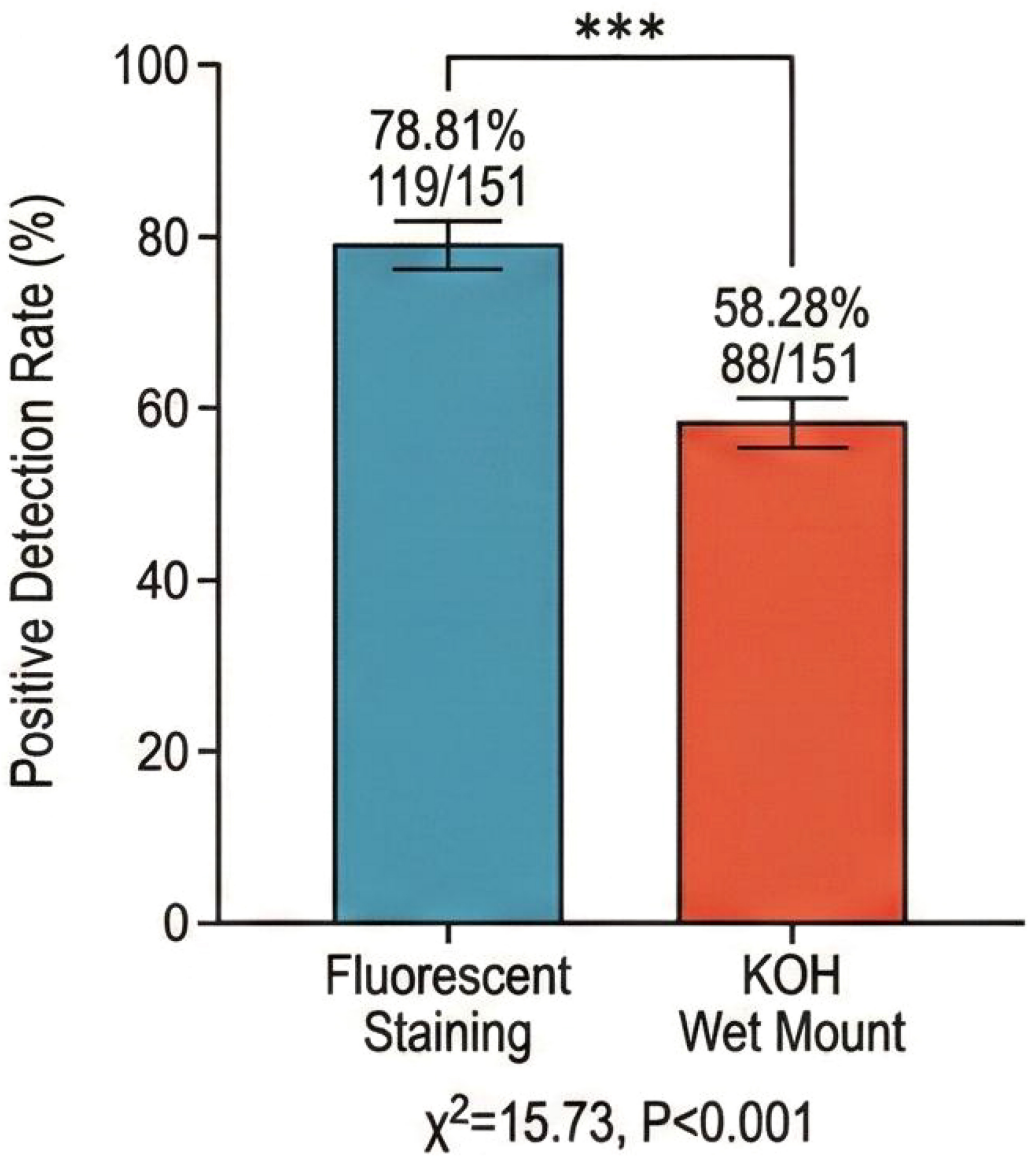
Bar chart comparing positive detection rates between fluorescent staining (78.81%) and KOH wet mount (58.28%) methods. Error bars represent 95% confidence intervals. ***P < 0.001 by McNemar’s test.

The fluorescent staining method demonstrated a significantly higher overall positive detection rate compared to the traditional KOH wet mount method. Of the 151 specimens examined, 119 (78.81%) were positive by fluorescent staining versus 88 (58.28%) by KOH wet mount, representing an absolute difference of 20.53 percentage points (McNemar’s test χ^2^ = 15.73, *P* < 0.001). The concordance between the two methods is illustrated in Table 2. Among the 119 fluorescent-positive cases, 85 (71.4%) were also positive by KOH, while 34 (28.6%) were detected exclusively by fluorescent staining. Conversely, only 3 specimens (2.0%) were positive by KOH alone while negative by fluorescent staining. All three of these discordant specimens were confirmed positive by fungal culture (two grew Trichophyton rubrum and one grew Candida albicans), indicating that fluorescent staining yielded false-negative results in these cases. The overall concordance rate between methods was 75.5% (114/151), with a kappa coefficient of 0.42 (95% CI: 0.29–0.55), indicating moderate agreement (Figure 2).

**TABLE 2:** Concordance between fluorescent staining and KOH wet mount results (*N* = 151).

KOH wet mount	Fluorescent staining		Total
	Positive	Negative	
Positive	85	3	88
Negative	34	29	63
Total	119	32	151

McNemar’s test χ^2^ = 15.73, *P* < 0.001; Kappa = 0.42 (95% CI: 0.29–0.55).

### 3.3 Diagnostic performance using fungal culture as reference

**FIGURE 3:**
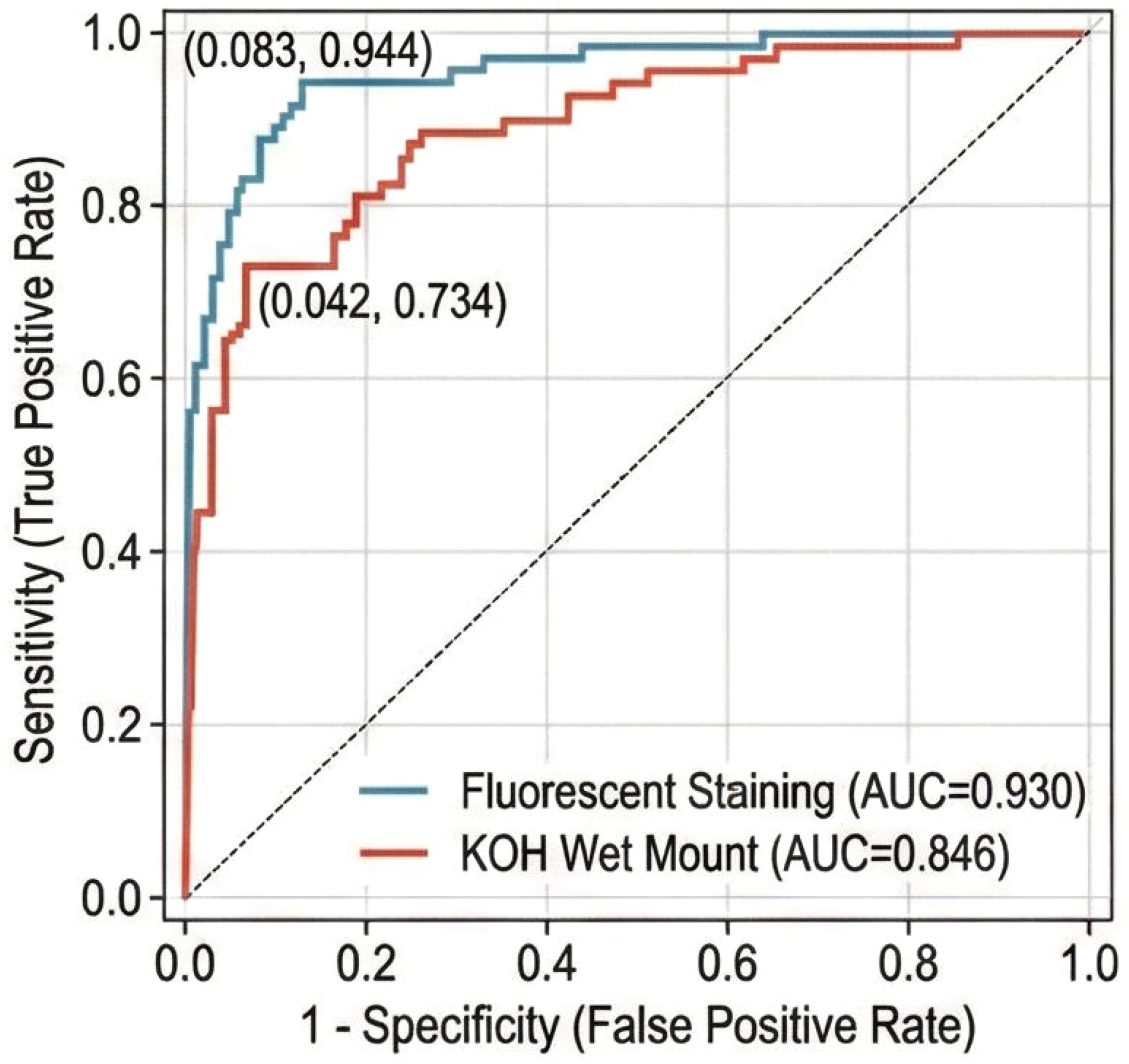
Receiver operating characteristic (ROC) curves comparing the diagnostic performance of fluorescent staining (AUC = 0.930) and KOH wet mount (AUC = 0.846) methods using fungal culture as the reference standard.

Fungal culture was positive in 124 of 151 specimens (82.1%), confirming true fungal infection. Among culture-positive cases, *Trichophyton rubrum* was the most frequently isolated species (*n* = 52, 41.9%), followed by Trichophyton mentagrophytes (*n* = 28, 22.6%), *Malassezia furfur* (*n* = 18, 14.5%), *Candida albicans* (*n* = 12, 9.7%), *Microsporum canis* (*n* = 8, 6.5%), and other species (*n* = 6, 4.8%). The diagnostic performance parameters for both methods are presented in Table 3. Using culture as the gold standard, fluorescent staining demonstrated significantly higher sensitivity (94.35%, 95% CI: 88.71–97.56%) compared to KOH wet mount (73.39%, 95% CI: 64.76–80.84%; *P* < 0.01 by McNemar’s test). The specificity of fluorescent staining (91.67%, 95% CI: 73.00–98.97%) was slightly lower than that of KOH (95.83%, 95% CI: 78.88–99.89%), though this difference did not reach statistical significance (*P* = 0.53). The positive predictive value was 98.32% for fluorescent staining versus 98.86% for KOH, while the negative predictive value was 75.86% for fluorescent staining versus 41.07% for KOH. Overall diagnostic accuracy was 93.38% for fluorescent staining compared to 77.48% for KOH wet mount (*P* < 0.001) (Figure 3).

**TABLE 3:** Diagnostic performance of fluorescent staining and KOH wet mount using fungal culture as reference standard.

Parameter	Fluorescent staining	KOH wet mount	*P*-value
Sensitivity, % (95% CI)	94.35 (88.71–97.56)	73.39 (64.76–80.84)	<0.01
Specificity, % (95% CI)	91.67 (73.00–98.97)	95.83 (78.88–99.89)	0.53
PPV, % (95% CI)	98.32 (94.01–99.75)	98.86 (93.85–99.97)	0.71
NPV, % (95% CI)	75.86 (57.74–88.37)	41.07 (29.43–53.66)	<0.001
Accuracy, % (95% CI)	93.38 (88.14–96.80)	77.48 (69.95–83.90)	<0.001

PPV, positive predictive value; NPV, negative predictive value; CI, confidence interval.

### 3.4 Subgroup analysis by specimen type

**FIGURE 4:**
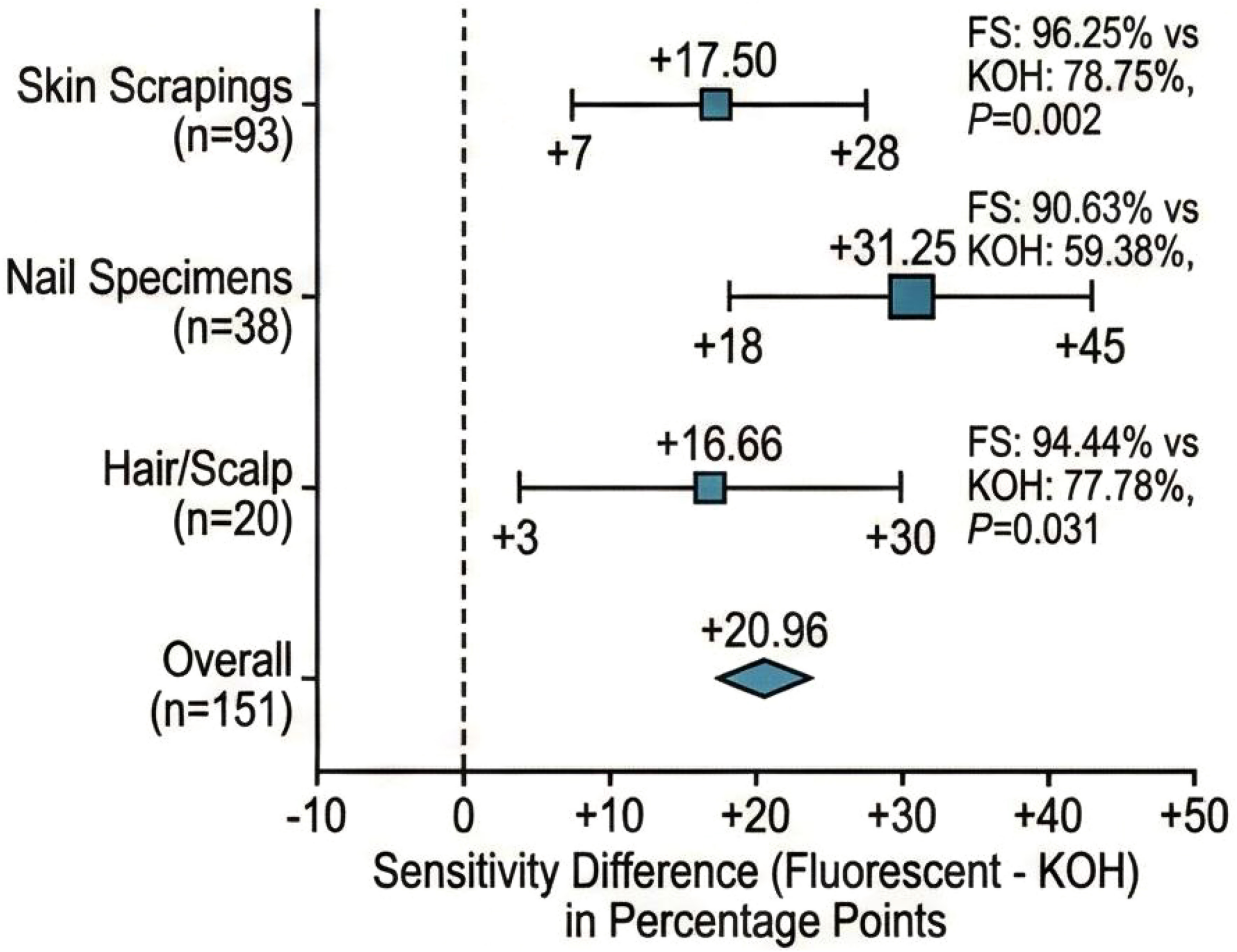
Forest plot showing the sensitivity difference (fluorescent staining minus KOH) with 95% confidence intervals stratified by specimen type. The dashed vertical line represents no difference (0%). All subgroups show significantly higher sensitivity with fluorescent staining.

Subgroup analysis revealed differential diagnostic performance across specimen types (Table 4). For skin scrapings (*n* = 93), fluorescent staining achieved sensitivity of 96.25% versus 78.75% for KOH (*P* = 0.002). For nail specimens (*n* = 38), the sensitivity advantage of fluorescent staining was even more pronounced (90.63% vs. 59.38%, *P* < 0.001). For hair and scalp specimens (*n* = 20), fluorescent staining demonstrated sensitivity of 94.44% compared to 77.78% for KOH (*P* = 0.031). The specificity remained comparable between methods across all specimen types. The absolute difference in sensitivity between methods was greatest for nail specimens (31.25 percentage points), followed by skin (17.50 percentage points) and hair/scalp (16.66 percentage points) (Figure 4).

**TABLE 4:** Diagnostic performance by specimen type.

Specimen type	n	FS sensitivity (%)	KOH sensitivity (%)	*P*-value
Skin scrapings	93	96.25	78.75	0.002
Nail specimens	38	90.63	59.38	<0.001
Hair/scalp	20	94.44	77.78	0.031

FS, fluorescent staining; KOH, potassium hydroxide.

### 3.5 Risk factors for missed diagnosis by KOH wet mount

**FIGURE 5:**
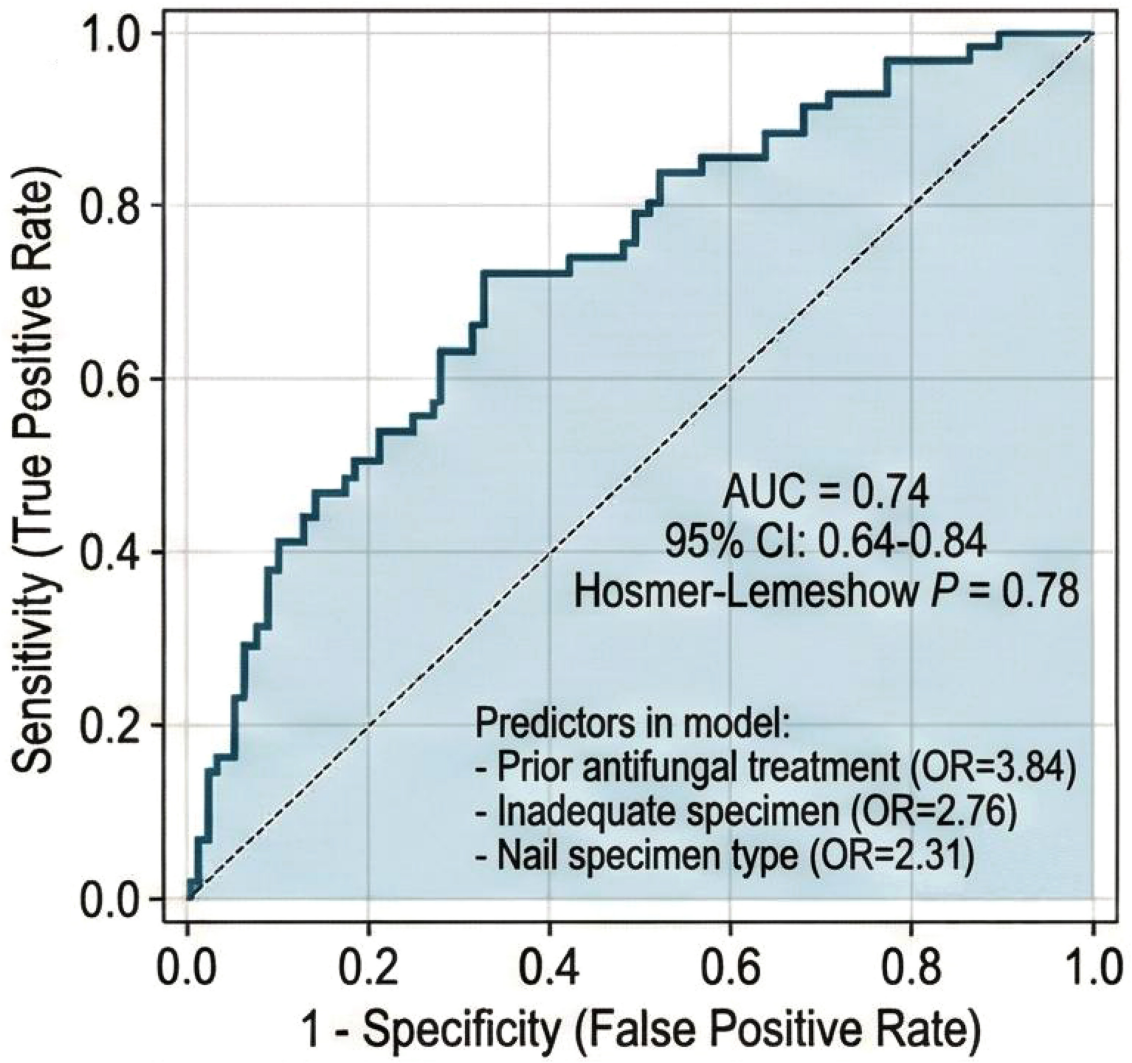
ROC curve for the multivariate logistic regression model predicting missed diagnosis by KOH wet mount. The area under the curve (AUC) was 0.74 (95% CI: 0.64–0.84), indicating acceptable discriminative ability.

Among the 124 culture-confirmed cases, 33 (26.6%) were missed by KOH wet mount examination while being correctly identified by fluorescent staining. Univariate analysis of factors associated with KOH-missed diagnosis is presented in Table 5. Factors significantly associated with missed diagnosis in univariate analysis included prior antifungal treatment (43.5% vs. 18.7%, *P* = 0.012), inadequate or borderline specimen adequacy (45.5% vs. 21.6%, *P* = 0.008), nail specimen type (42.1% vs. 22.6%, *P* = 0.038), and symptom duration > 12 weeks (36.4% vs. 21.8%, *P* = 0.089). Multivariate logistic regression analysis (Table 6) identified three independent risk factors for missed diagnosis: prior antifungal treatment (adjusted OR = 3.84, 95% CI: 1.62–9.11, *P* = 0.002), inadequate or borderline specimen adequacy (adjusted OR = 2.76, 95% CI: 1.18–6.45, *P* = 0.019), and nail specimen type (adjusted OR = 2.31, 95% CI: 1.04–5.13, *P* = 0.039). The Hosmer-Lemeshow test indicated good model fit (χ^2^ = 4.83, *P* = 0.78), and the area under the ROC curve for the predictive model was 0.74 (95% CI: 0.64–0.84) (Figure 5).

**TABLE 5:** Univariate analysis of factors associated with missed diagnosis by KOH wet mount (*N* = 124 culture-positive cases).

Variable	KOH-missed (*n* = 33)	KOH-detected (*n* = 91)	*P*-value
Age (years), mean ± SD	45.2 ± 16.1	43.1 ± 14.9	0.49
Male sex, n (%)	19 (57.6)	50 (54.9)	0.79
Prior antifungal treatment, n (%)	10 (30.3)	11 (12.1)	0.012
Inadequate/borderline specimen, n (%)	15 (45.5)	15 (16.5)	0.001
Nail specimen type, n (%)	13 (39.4)	19 (20.9)	0.038
Symptom duration > 12 weeks, n (%)	12 (36.4)	22 (24.2)	0.18

**TABLE 6:** Multivariate logistic regression analysis of independent risk factors for missed diagnosis.

Variable	Adjusted OR	95% CI	*P*-value
Prior antifungal treatment	3.84	1.62–9.11	0.002
Inadequate/borderline specimen	2.76	1.18–6.45	0.019
Nail specimen type	2.31	1.04–5.13	0.039

OR, odds ratio; CI, confidence interval. Hosmer-Lemeshow χ^2^ = 4.83, *P* = 0.78; AUC = 0.74 (95% CI: 0.64–0.84).

## 4 Discussion

This study provides several novel contributions to the existing evidence base. First, using multivariate regression analysis, we quantified three modifiable independent risk factors for KOH diagnostic failure—prior antifungal treatment (OR = 3.84), inadequate specimen quality (OR = 2.76), and nail specimen type (OR = 2.31)—all of which have direct implications for clinical practice protocols. Second, we demonstrate that the diagnostic gain of fluorescent staining varies substantially by specimen type, with the absolute sensitivity improvement being greatest for nail specimens (31.25 percentage points) compared to skin scrapings (17.50 percentage points) and hair/scalp (16.66 percentage points), providing stratified evidence to guide resource allocation. Third, methodologically, our paired design with independent blinded reading and a robust reference standard (culture with species-level identification) minimizes the information bias present in many prior studies. The remaining confirmatory findings—such as the overall superiority of fluorescent staining over KOH in sensitivity and comparable specificity—are consistent with previous reports and are summarized briefly below.

The fluorescent staining method demonstrated a positive detection rate of 78.81% versus 58.28% for KOH, representing a statistically significant and clinically meaningful improvement of 20.53 percentage points. When validated against fungal culture as the reference standard, fluorescent staining achieved a sensitivity of 94.35% compared to 73.39% for KOH, while maintaining comparable specificity. These findings align with and extend previous investigations and underscore the enhanced detection capabilities of fluorescent techniques (15).

The observed sensitivity advantage of fluorescent staining in our study is consistent with the mechanistic basis of this technique. Calcofluor white binds specifically to chitin and β-glucans in fungal cell walls, producing bright fluorescence that creates stark contrast against the dark background, thereby facilitating detection of even sparse fungal elements that might be overlooked in unstained preparations (16). Our finding of 94.35% sensitivity is comparable to the 97% sensitivity reported by Mourad et al. in their evaluation of calcofluor white staining for dermatophytosis of hair and nails (15), and exceeds the 85% sensitivity documented in earlier studies using different fluorescent reagent formulations (16). The slightly lower sensitivity in our cohort may reflect the heterogeneous nature of our sample, which included various specimen types and clinical presentations rather than focusing on a single disease entity. Importantly, the high sensitivity was achieved without substantial compromise in specificity (91.67%), indicating that fluorescent staining reliably identifies true infections while generating few false-positive results (17, 18). Although fluorescent staining demonstrated superior sensitivity overall, three culture-confirmed cases were missed by this method. Possible explanations for false-negative results with fluorescent staining include: (1) low fungal burden with sparse organisms that may be missed even with enhanced contrast; (2) sampling variability between the portions of specimen allocated to different methods, particularly in heterogeneous lesions where fungal distribution is uneven; (3) quenching of fluorescence due to excessive background debris or autofluorescence from keratin or other tissue components; and (4) technical factors such as inadequate reagent mixing, insufficient clearing time, or suboptimal microscope focus. Notably, the three cases missed by fluorescent staining were all correctly identified by KOH examination, suggesting that the two methods may be complementary in certain low-burden scenarios. These observations underscore the importance of clinical correlation and, when clinically warranted, repeat sampling even when initial fluorescent staining is negative.

The KOH wet mount sensitivity of 73.39% observed in our study falls within the broad range reported in the literature, which spans from 33.7 to 93% depending on the clinical context and methodological factors (19). A meta-analysis by Velasquez-Agudelo and Cardona-Arias examining diagnostic methods for onychomycosis reported a pooled KOH sensitivity of 61%, which is lower than our finding but may reflect differences in specimen types included (20). The variability in KOH sensitivity across studies underscores the technique’s dependence on multiple factors including specimen quality, clearing time, examiner expertise, and microscope quality. In contrast, the enhanced contrast provided by fluorescent staining appears to reduce this variability by making fungal elements more conspicuous regardless of background conditions (21). Our finding of moderate agreement (kappa = 0.42) between methods indicates that they capture overlapping but not identical fungal populations, with fluorescent staining detecting a substantial proportion of cases missed by KOH alone (22).

Subgroup analysis by specimen type revealed that the sensitivity advantage of fluorescent staining was most pronounced for nail specimens, where KOH sensitivity dropped to 59.38% compared to 90.63% for fluorescent staining. This observation is clinically relevant given the known diagnostic challenges associated with onychomycosis, where thick keratinous material can obscure fungal elements and hinder adequate clearing with KOH (23). Previous studies have similarly documented lower KOH sensitivity for nail specimens compared to skin scrapings, with some investigators reporting sensitivity as low as 33.7% for conventional microscopy in onychomycosis (24). The substantial improvement in detection achieved with fluorescent staining for nail specimens suggests that this technique should be particularly prioritized in the diagnostic workup of suspected onychomycosis, where accurate diagnosis is essential to guide the prolonged and potentially hepatotoxic systemic antifungal therapy often required for cure (25). It should be noted that we used 10% KOH for all specimen types, whereas 20% KOH is often recommended for thick nail clippings to achieve more complete clearing. The use of 10% KOH may have resulted in suboptimal clearing of nail specimens, potentially contributing to the lower KOH sensitivity observed in this subgroup (59.38%) and partially explaining why nail specimen type emerged as an independent risk factor for missed diagnosis. This methodological consideration further supports the preferential use of fluorescent staining for nail specimens, as the fluorescent technique does not rely on complete keratin clearing for fungal element visualization.

The systematic identification and quantification of independent risk factors for missed diagnosis by KOH examination represents a key novelty of our work.. Multivariate analysis revealed that prior antifungal treatment was the strongest predictor of diagnostic failure (OR = 3.84), followed by inadequate specimen quality (OR = 2.76) and nail specimen type (OR = 2.31) (26). The association between prior antifungal treatment and missed diagnosis is biologically plausible, as antifungal agents reduce fungal burden and may alter cell wall integrity, rendering remaining organisms more difficult to detect with unstained preparations. This finding has practical implications for clinical practice, suggesting that patients who present after unsuccessful self-treatment with topical antifungals—a common scenario—may particularly benefit from fluorescent staining or repeated sampling after a washout period (27).

The association between specimen adequacy and diagnostic accuracy highlights the critical importance of proper sampling technique. Inadequate specimens reduce the probability of capturing fungal elements regardless of the examination method employed, but our data suggest that this effect is more pronounced with KOH than with fluorescent staining. This observation may reflect the ability of fluorescent techniques to detect smaller quantities of fungal material due to their enhanced signal-to-noise ratio (28). Nevertheless, optimal specimen collection remains fundamental to diagnostic success, and our findings support continued emphasis on training and quality assurance in this area. The development of standardized adequacy criteria and real-time feedback mechanisms could potentially improve specimen quality and reduce the need for repeat sampling.

From a clinical implementation perspective, the superior diagnostic performance of fluorescent staining must be weighed against practical considerations including equipment requirements, reagent costs, and training needs. Fluorescence microscopy requires specialized equipment with appropriate excitation and emission filters, which represents a capital investment beyond that required for standard light microscopy (15). However, the incremental cost per test is modest, as calcofluor white reagent is inexpensive and shelf-stable. Moreover, the improved sensitivity translates to reduced need for repeat testing, earlier initiation of appropriate therapy, and avoidance of unnecessary treatments in patients without true infection. A formal cost-effectiveness analysis would be valuable to quantify the economic impact of implementing fluorescent staining as a first-line diagnostic approach in different clinical settings.

Several limitations of our study warrant acknowledgment. First, the retrospective design introduces potential selection bias, as patients were not randomly assigned to diagnostic methods but rather underwent both tests as part of routine clinical care. Second, the single-center setting may limit generalizability to other populations or practice environments. Third, we relied on fungal culture as the reference standard, which itself has imperfect sensitivity (reported at 50–70% in various studies) and may have led to misclassification of some true-positive microscopy results as false positives (29). Fifth, the relatively small sample size limited our power to detect associations with smaller effect sizes and precluded robust subgroup analyses by specific clinical diagnoses or fungal species. Sixth, yeast species identification was performed using phenotypic methods (CHROMagar differential medium, microscopic morphology, and API 20C AUX biochemical profiling) rather than molecular techniques such as ITS region sequencing or MALDI-TOF mass spectrometry. While these conventional methods are standard in many clinical mycology laboratories, they may have reduced discriminatory power for closely related species within complexes such as the *Candida parapsilosis* group or certain *Malassezia* species, potentially leading to misidentification. This limitation should be considered when interpreting species-specific findings. Sixth, yeast species identification was performed using phenotypic methods (CHROMagar differential medium, microscopic morphology, and API 20C AUX biochemical profiling) rather than molecular techniques such as ITS region sequencing or MALDI-TOF mass spectrometry. While these conventional methods are standard in many clinical mycology laboratories, they may have reduced discriminatory power for closely related species within complexes such as the Candida parapsilosis group or certain Malassezia species, potentially leading to misidentification. This limitation should be considered when interpreting species-specific findings. Future studies employing molecular identification methods would be valuable to confirm species-level epidemiological patterns.

Future research directions include prospective multicenter studies with larger and more diverse populations, cost-effectiveness analyses comparing different diagnostic algorithms, and investigations of alternative fluorescent reagents or combination approaches that might further enhance diagnostic performance. The development of point-of-care fluorescence devices suitable for office-based use could potentially expand access to this technology in primary care and resource-limited settings. Additionally, studies examining the clinical impact of improved diagnosis on patient outcomes, including treatment success rates, time to cure, and quality of life, would provide valuable evidence to guide practice recommendations and resource allocation decisions.

In conclusion, this study demonstrates that fluorescent staining provides significantly superior diagnostic performance compared to traditional KOH wet mount examination for superficial fungal skin infections, with a 21 percentage point improvement in sensitivity while maintaining high specificity. The sensitivity advantage is particularly pronounced for nail specimens, which pose the greatest diagnostic challenge with conventional methods. Prior antifungal treatment, inadequate specimen quality, and nail specimen type are independent risk factors for missed diagnosis with KOH examination. These findings support the adoption of fluorescent staining as a primary diagnostic tool for suspected superficial mycoses, especially in patients with prior treatment exposure or when dealing with nail specimens. Implementation of fluorescent staining protocols has the potential to reduce diagnostic delays, minimize inappropriate treatments, and improve outcomes for patients with superficial fungal infections.

## Data Availability

The raw data supporting the conclusions of this article will be made available by the authors, without undue reservation.

## References

[1] Bongomin F Gago S Oladele R Denning D. Global and multi-national prevalence of fungal diseases—estimate precision. *J Fungi*. (2017) 3:57. 10.3390/jof3040057 29371573 PMC5753159

[2] Hay RJ. Fungal Infections of the Skin and Subcutaneous Tissue. In: Kibbler CC Barton R Gow NAR editors. *Oxford Textbook of Medical Mycology.* Oxford: Oxford University Press (2018). p. 145–53.

[3] Ameen M. Epidemiology of superficial fungal infections. *Clin Dermatol*. (2010) 28:197–201. 10.1016/j.clindermatol.2009.12.005 20347663

[4] Benedict K Gold JAW Wu K Lipner SR. High frequency of self-diagnosis and self-treatment in a nationally representative survey about superficial fungal infections in adults-united states, 2022. *J Fungi.* . (2022) 9:19. 10.3390/jof9010019 36675840 PMC9860956

[5] Hill RC Caplan AS Elewski B Gold JAW Lockhart SR Smith DJ et al. Expert panel review of skin and hair dermatophytoses in an era of antifungal resistance. *Am J Clin Dermatol.* (2024) 25:359–89. 10.1007/s40257-024-00848-1 38494575 PMC11201321

[6] Shankland GS. Microscopy and Culture of Fungal Disease. In: Kibbler CC Barton R Gow NAR et al. editors. *Oxford Textbook of Medical Mycology.* Oxford: Oxford University Press (2018). p. 283–8.

[7] Pan L Yang B Huang J Hu L-H Tao Y-F Lu M-Q et al. Fluorescent staining method vs KOH wet mount method in the diagnosis of finger (toe) nail infection. *Chin J Microecol.* (2022) 34:220–2, 227. 10.13381/j.cnki.cjm.202202018

[8] Karaman BFO Açıkalın A Ünal İ Aksungur VL. Diagnostic values of KOH examination, histological examination, and culture for onychomycosis: a latent class analysis. (2019) 58:319–24. 10.1111/ijd.14255 30246397

[9] Mendonça A Santos H Franco-Duarte R Sampaio P. Fungal infections diagnosis - past, present and future. *Res Microbiol*. (2022) 173:103915. 10.1016/j.resmic.2021.103915 34863883 PMC8634697

[10] Bao F Fan Y Sun L Yu Y Wang Z Pan Q et al. Comparison of fungal fluorescent staining and ITS rDNA PCR-based sequencing with conventional methods for the diagnosis of onychomycosis. *J Eur Acad Dermatol Venereol*. (2018) 32:1017–21. 10.1111/jdv.14843 29405481 PMC6001524

[11] Afshar P Larijani LV Rouhanizadeh H. A comparison of conventional rapid methods in diagnosis of superficial and cutaneous mycoses based on KOH. *Iran J Microbiol.* (2018) 10:433–40. 30873272 PMC6414738

[12] Howell SA. Dermatopathology and the Diagnosis of Fungal Infections. *Br J Biomed Sci*. (2023) 80:11314. 10.3389/bjbs.2023.11314 37351018 PMC10282148

[13] Rathi VM Murthy SI Mitra S Yamjala B Mohamed A Sharma S. Masked comparison of trypan blue stain and potassium hydroxide with calcofluor white stain in the microscopic examination of corneal scrapings for the diagnosis of microbial keratitis. *Indian J Ophthalmol*. (2021) 69:2457–60. 10.4103/ijo.IJO_3685_20 34427244 PMC8544045

[14] Zhang S Chen Z Deng Z Fu P Yu Y Zhuang Q et al. The value of calcofluor white in the diagnosis of invasive fungal diseases. *Diagn Microbiol Infect Dis*. (2024) 108:116186. 10.1016/j.diagmicrobio.2024.116186 38278003

[15] Mourad B Ismail M Hawwam S Msseha M Hassan R. Evaluation of the efficacy of fluorescent staining and chicago sky blue staining as methods for diagnosis of dermatophytosis in hair and nails. *Clin Cosmet Investig Dermatol*. (2019) 12:751–8. 10.2147/CCID.S215661 31632123 PMC6790344

[16] Kirani KR Chandrika VS. Efficacy of in-house fluorescent stain for fungus. *Indian J Pathol Microbiol*. (2017) 60:57–60. 10.4103/0377-4929.200049 28195092

[17] Dass SM Vinayaraj EV Pavavni K Pallam A Rao MS. Comparison of KOH. *Indian J Microbiol Res*. (2015) 2:148. 10.5958/2394-5478.2015.00004.7

[18] Pihet M Le Govic Y. Reappraisal of conventional diagnosis for dermatophytes. *Mycopathologia*. (2017) 182:169–80. 10.1007/s11046-016-0071-y 27718160

[19] Gupta AK Hall DC Cooper EA Ghannoum MA. Diagnosing onychomycosis: what’s new? *J Fungi*. (2022) 8:464. 10.3390/jof8050464 35628720 PMC9146047

[20] Velasquez-Agudelo V Cardona-Arias JA. Meta-analysis of the utility of culture, biopsy, and direct KOH examination for the diagnosis of onychomycosis. *BMC Infect Dis*. (2017) 17:166. 10.1186/s12879-017-2258-3 28222676 PMC5320683

[21] Falotico JM Lipner SR. Updated perspectives on the diagnosis and management of onychomycosis. *Clin Cosmet Investig Dermatol*. (2022) 15:1933–57. 10.2147/CCID.S362635 36133401 PMC9484770

[22] Warnnissorn P Sawatdiwithayayong J Surit P. Efficacy and rapidity of potassium hydroxide mount and modified chicago sky blue 6b stain with potassium hydroxide in fungal keratitis detection. *Korean J Ophthalmol*. (2024) 38:98–104. 10.3341/kjo.2023.0106 38351488 PMC11016681

[23] Jung MY Shim JH Lee JH Lee JH Yang JM Lee DY et al. Comparison of diagnostic methods for onychomycosis, and proposal of a diagnostic algorithm. *Clin Exp Dermatol*. (2015) 40:479–84. 10.1111/ced.12593 25683452

[24] Gupta AK Daigle D Carviel JL. The role of fungi in dermatology: a review. *J Clin Aesthet Dermatol.* (2017) 10:28–40.

[25] Lipner SR Scher RK. Onychomycosis: clinical overview and diagnosis. *J Am Acad Dermatol*. (2019) 80:835–51. 10.1016/j.jaad.2018.03.062 29959961

[26] Haghani I Shokohi T Hajheidari Z Khalilian A Aghili SR. Comparison of diagnostic methods in the evaluation of onychomycosis. *Mycopathologia*. (2013) 175:315–21. 10.1007/s11046-013-9620-9 23371413

[27] Wei LW Qiao JJ. Mini-Review: the diagnostic methods of tinea capitis. *Mycopathologia*. (2023) 188:563–9. 10.1007/s11046-023-00731-3 37067665

[28] Hasan S Gupta P Shukla D Banerjee GA. Comparison between potassium hydroxide (KOH) microscopy and culture for the detection of post-COVID-19 rhino-orbital-cerebral mucormycosis. *Cureus*. (2023) 15:e47707. 10.7759/cureus.47707 38022015 PMC10674886

[29] Leung AK Barankin B Lam JM Leong KF Hon KL. Tinea pedis: an updated review. *Drugs Context.* (2023) 12:2023–5. 10.7573/dic.2023-5-1 37415917 PMC10321471

